# Balanced chromosomal rearrangement in a partner revealed after Preimplantation Genetic Testing for Aneuploidies (PGT-A)

**DOI:** 10.5935/1518-0557.20220058

**Published:** 2023

**Authors:** Ianaê Ceschin, Taccyanna M. Ali, Cristina V. Carvalho, Caique Martins, Adriana Bos-Mikich, Gabriella M. Andrade, Nilo Frantz

**Affiliations:** 1 Igenomix Brazil - Laboratório de Genética e Medicina Reprodutiva, São Paulo, Brazil; 2 Federal University of Rio Grande do Sul, Porto Alegre, Brazil; 3 Nilo Frantz Reproductive Medicine, Porto Alegre, Brazil

**Keywords:** chromosomal structural rearrangement, reciprocal translocation, embryo selection

## Abstract

In general population, it is estimated that 1/560 -1/1100 of the individuals are carriers of a balanced structural alteration and, in general, do not present an abnormal phenotype. For patients who have balanced rearrangements, a family planning alternative is to perform an In Vitro Fertilization (IVF) cycle with the embryonic analysis by Preimplantation Genetic Testing for Chromosomal Structural Rearrangements (PGT-SR). This test aims to reduce the time to obtain a healthy chromosomally pregnancy, to minimize the risk of miscarriage and a live birth with a chromosomopathy. The present work reports a case in which the couple had a history of implantation failure and biochemical pregnancy. They had not performed the karyotype exam to verify the parents’ chromosomal content. After two embryo transfers without achieving pregnancy, the couple was directed to the Preimplantation Genetic Testing for Aneuploidies (PGT-A). The result presented in PGT-A in the couple’s first cycle using the embryo selection technique showed recurrent segmental aneuploidies the trophectoderm biopsies. The couple was given genetic counselling, and they decided to investigate their karyotype, which showed a balanced chromosomal rearrangement in one of the parents. With this investigation and genetic counselling, it was possible to apply the correct embryonic analysis strategy, which contributed to a healthy pregnancy and birth with a living child.

## INTRODUCTION

In the general population, balanced chromosomal rearrangements (e.g., translocations, inversions, and insertions) are estimated to be about 1/560 - 1/1100 (Jacobs *et al.*, 1992). When couples with infertility undergo genetic testing, especially in men, the incidence of abnormal karyotype increases to 0.6% (Mau-Holzmann, 2005).

Reciprocal translocations often involve autosomal chromosomes, but they may also involve sex chromosomes. Genetic material is swapped between two non-homologous chromosomes in reciprocal translocations. Patients with a balanced chromosomal rearrangement usually present a normal phenotype but a higher risk of producing abnormal gametes and embryos when compared to non-carriers (Campana *et al.*, 1986; Stern *et al.*, 1999; Lledó *et al.*, 2010). Some unbalanced chromosomal rearrangements are closely related to severe congenital diseases such as mental retardation, malformations, and fetal death, but the phenotype will depend on the abnormal chromosomal segment involved (Yin *et al.*, 2017). In approximately 2-5% of couples with recurrent miscarriage, one of the partners is affected by a chromosomal translocation (Brezina *et al.*, 2012). However, empirical and theoretical risks of producing unbalanced gametes are different, and they can differ in terms of the gender of the translocation carrier (Coco, 2018).

For those who are carriers of balanced chromosomal rearrangement, an alternative could be an In Vitro Fertilization (IVF) cycle with Preimplantation Genetic Testing for Chromosomal Structural Rearrangements (PGT-SR). Whereas Preimplantation Genetic Testing for Aneuploidies (PGT-A) is a screening tool for chromosomal abnormalities that arise spontaneously, PGT-SR is a targeted test performed when known balanced chromosomal rearrangements are present in parental genomes (Viotti, 2020). PGT-A is recommended for advanced maternal age cases, implantation failures, and recurrent miscarriage (Ormond *et al.*, 2018). PGT-SR requires a personalized review of parental karyotypes, as the resulting cohort of embryos is tested for instances of recombination producing unbalanced chromosomal configurations in at-risk regions (Viotti, 2020), and it can detect chromosomal abnormalities > 6Mb while PGT-A can detect alterations >10 Mb (García-Pascual *et al.*, 2020). With these techniques, it is possible to select balanced/euploid embryos for transfer to the maternal uterus to improve the chances of pregnancy. Both methods aim to decrease the time to obtain a healthy pregnancy and reduce the risk of miscarriage (Fischer *et al.*, 2010).

## CASE REPORT

A 34-year-old female and a 35-year-old-male came to the clinic to achieve a pregnancy, and they had no family history of genetic diseases. Regarding male evaluation, in the sperm analysis, the macroscopic parameters found were in the normal range according to the World Health Organization (WHO) (WHO, 2010). However, in the microscopic parameters, the percentage of progressively motile sperm was 16%. The couple had a previous history of two embryo transfers that did not result in a viable pregnancy. In the first attempt, there was implantation failure, and in the second, biochemical pregnancy. The couple decided to undergo a first IVF cycle with PGT-A to increase the chances of pregnancy. This case report was not submitted to the ethics committee because only clinical data were used in this study. The patients informed consent to anonymous use of their clinical data in scientific studies before the procedure. This informed consent was reviewed and approved by the Brazilian Health Regulatory Agency (ANVISA).

### IVF cycles, PGT-A, and PGT-SR

In the first IVF treatment with PGT-A, the patient had 8 metaphase II (MII) oocytes, of which 7 fertilized normally and 5 embryos made it to the blastocyst stage. Still, only 4 had the quality to perform the trophectoderm biopsy procedure. Thus two embryos were biopsied on day 05 of development and the others on day 06. Trophectoderm cells were analyzed by PGT-A using Next Generation Sequencing (NGS) technology according to the protocol of García-Pascual and colleagues (García-Pascual *et al.*, 2020) at the Igenomix Brazil laboratory. Sequencing was performed with an Ion S5 System (Thermo Fisher Scientific, USA). Raw sequence data in FASTQ format were aligned to the hg19 human reference genome implemented in Ion Reporter software (Thermo Fisher Scientific, USA).

**Figure 1:**
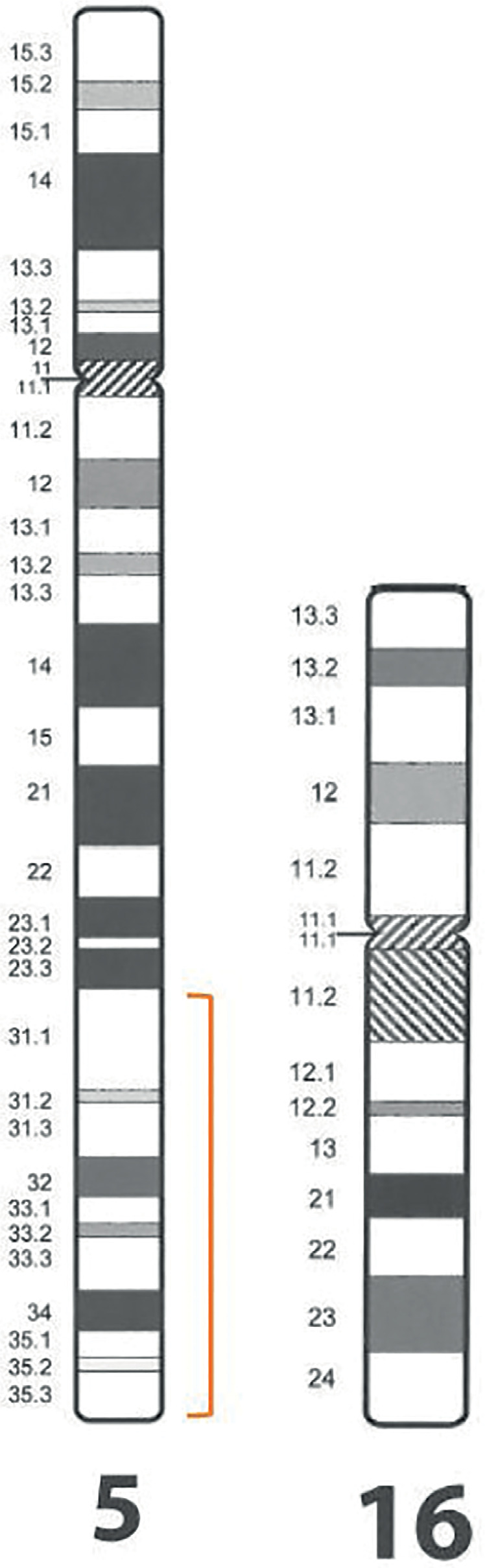
Chromosomal segments involved in the reciprocal translocation in the male patient. Karyotyping showed a balanced translocation between 5 and 16 chromosomes - 46,XY,t(5;16)(q31.1;p13.3). The figure was adapted from International System for Human Cytogenetic Nomenclature (ISCN) 2016 (McGowan-Jordan *et al*., 2016).

In the first IVF cycle, with PGT-A, three of four embryos showed segmental aneuploidies - a duplication of the long arm (q) of chromosome 5 (+5q) and a deletion of the short arm (p) of chromosome 16 (-16p). Only one blastocyst was considered euploid/balanced. After PGT-A results showed segmental alterations, the couple received medical advice and genetic counseling. G-banding karyotype analysis of peripheral blood lymphocytes was performed in the couple, and reciprocal translocation involving chromosomes 5 and 16 (46,XY,t(5;16)(q31.1;p13.3)) was revealed in the male. The chromosomal segments involved in rearrangement are shown in Figure 1.

After genetic counseling, the couple decided to perform another IVF cycle, now with PGT-SR, which allowed chromosomal structural rearrangements detection with greater precision than PGT-A.

**Figure 2:**
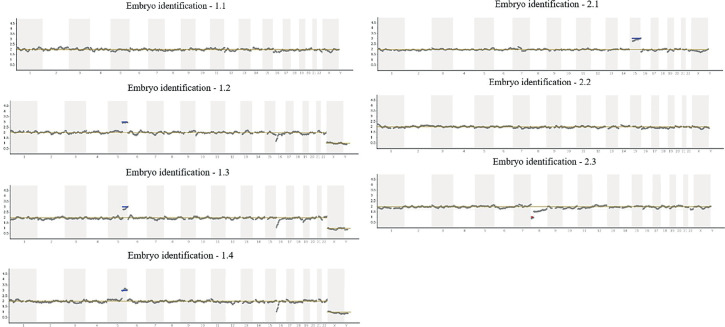
The NGS graphs showing the chromosomal profile from the trophectoderm biopsies.

In the second FIV cycle performed at the same clinic, the patient had 7 metaphase II (MII) oocytes, 7 fertilized normally, and 3 embryos made it to the blastocyst stage. They had the quality to perform the trophectoderm biopsy procedure. Regarding the genetic analysis, now with PGT-SR, they had one euploid/balanced embryo, one embryo with trisomy 15 (+15), and one embryo with a deletion in the short arm (p) of chromosome 8 (-8p). The results are summarised in Table 1, and the NGS graphs showing the chromosomal profile are shown in Figure 2. The couple underwent embryo thaw cycles followed by transfer to the uterine cavity.

**Table 1:** Summary of genetic results from two IVF cycles with PGT-A or PGT-SR.

Embryo identification	Biopsy day	Blastocyst grade	Type of PGT	Result
**1.1**	D5	3AA	PGT-A	46,XX
**1.2**	D5	3AA	PGT-A	46,XY(+5q,-16p)
**1.3**	D6	3AA	PGT-A	46,XY(+5q,-16p)
**1.4**	D6	3AA	PGT-A	46,XY(+5q,-16p)
**2.1**	D5	3AA	PGT-SR	47,XX,+15
**2.2**	D5	3AA	PGT-SR	46,XX
**2.3**	D5	3AB	PGT-SR	46,XX(-8p)

Legend: p- short arm of the chromosome; q-long arm of the chromosome.

The euploid/balanced embryo (identified as 1.1) was transferred from the first IVF cycle with PGT-A did not implant. The couple underwent another endometrial preparation followed by euploid/balanced embryo transfer (identified as 2.2) from the second IVF cycle with PGT-SR. This embryo successfully generated the clinical pregnancy and resulted in a female baby weighing 2,220 kg at birth.

## DISCUSSION

The present case describes a couple with reproductive issues, being the male, a carrier of a balanced chromosomal rearrangement revealed after an IVF cycle with PGT-A analysis. In our case report, the first euploid/balanced embryo transfer (embryo 1.1) resulted in no implantation. However, the second euploid/balanced embryo transfer (embryo 2.2) resulted in the birth of a healthy baby. Aneuploidy, in general, is the leading cause of miscarriage and implantation failures (Hassold *et al.*, 1996). Nevertheless, there are many potential reasons why euploid blastocysts do not always result in a viable pregnancy, such as endometrial receptivity, uterine factors, and embryo-endometrial synchrony (Meldrum & de Ziegler, 2016). Thus, these factors, which we call “embryo-endometrium crosstalk” could explain the implantation failure in the couple’s first IVF with the PGT cycle.

In this case report, about 40% of the embryos presented two segmental aneuploidies - a duplication of the long arm (q) of chromosome 5 (+5q) and a deletion of the short arm (p) of chromosome 16 (-16p). In the literature, different outcomes can be according to the kind of segregation, such as adjacent I, adjacent II, alternating, 3: 1, and 4:0. Gametes from alternate segregation are normal or balanced; thus, it is the only mode that leads to gametes with a complete genetic complement. All other modes can be malsegregation (Gardner & Sutherland, 2011). Conceptions from adjacent-I gametes have trisomy (duplication) for one translocated segment and monosomy (deletion) for the other. Adjacent-II conceptions have trisomy for one centric segment and monosomy for the other. In 3:1 nondisjunction, two categories exist: either the two normal chromosomes of the quadrivalent plus one of the translocation chromosomes go together to one daughter cell (tertiary trisomy) or, rarely, the two translocation chromosomes and one of the normal chromosomes segregate (interchange trisomy). In 4:0 segregation, there is a double trisomy or double monosomy (Gardner & Sutherland, 2011).

In the case of adjacent segregation I, fertilization by a euploid gamete could lead to an embryo carrier of a deletion of the long arm (q) of chromosome 5 and a duplication of the long arm (q) of chromosome 16 (-5q; +16q). Another possibility of this type of segregation is a duplication of the long arm (q) of chromosome 5 and deletion of chromosome 16 (+5q, -16p) (Scriven *et al*., 1998).

For adjacent segregation II, if the gametes produced were fertilized by another euploid gamete, the embryos would present chromosomal abnormalities such as a duplication of the short arm (p) of chromosome 5 and deletion of the short arm (p) of chromosome 16 (+5p; -16p) or a deletion of the long arm (q) of chromosome 5 and duplication of the long arm (q) of chromosome 16 (-5q; +16q) (Scriven *et al*., 1998).

In the cohort of analyzed embryos, the presence of two embryos with whole or segmental chromosomal aneuploidy could be justified by the interchromosomal effect, but we could not determine their origin. In these cases, the structural alteration could cause an incorrect alignment of the chromosomes during the division and generate whole or segmental chromosomal aneuploidies in the embryos (Lejeune, 1963).

The G-banding karyotype analysis is essential because it can identify couples with an increased risk of generating aneuploid embryos, and it may help the decision and choose an appropriate assisted reproduction treatment (Foresta *et al.,* 2002; Hunt & Hassold, 2002), especially in cases that involve changing parameters in seminal analysis, such as low sperm concentration. Cytogenetic data for 447 couples referred for intra-cytoplasmic sperm injection ICSI treatment showed that 2.1% of the males with constitutional chromosomal aberrations, thus they have a greater risk of being carriers of chromosomal abnormality when compared to the general population (Meschede *et al*., 1998; Van Assche *et al.*, 1996). About 5% of male infertility cases can be attributed to chromosomal abnormalities (Pelzman & Hwang, 2021).

However, in this report, the G-banding karyotype analysis and the identification of balanced translocation were possible after the genetic embryo resulted in the PGT-A by NGS, which showed atypical chromosomal alterations leading to a suspicion of chromosomal rearrangement.

Assisted reproduction and genetic testing technologies continue to evolve and change. However, the NGS method, as well as other techniques, has its limitations. The genetic analysis method does not identify aneuploidy’s origin, whether mitotic or meiotic (ESHRE PGT-SR / PGT-A Working Group *et al.*, 2020). It is not possible to differentiate between an embryo with a euploid karyotype or a balanced rearrangement. Detecting fetal mosaicism is also challenging since the degree of mosaicism is estimated from the trophectoderm cells and not from embryoblast (García-Pascual *et al.*, 2020). Also, although balanced embryos should result in phenotypically normal births, the offspring will, later in life, encounter the same problems as their carrier parents (Viotti, 2020). Thus genetic counseling should be offered to all these families at risk.

## CONCLUSION

The present study showed a couple with infertility who resorted to assisted reproduction treatment technology without knowing the male was a carrier of a balanced rearrangement. PGT-A and PGT-SR are accurate analysis methods for detecting aneuploidies related to malsegregation. The work highlights the importance of performing the G-banding karyotype and genetic counseling for patients with structural rearrangements for family planning.
